# Do Participatory Learning and Action Women’s Groups Alone or Combined with Cash or Food Transfers Expand Women’s Agency in Rural Nepal?

**DOI:** 10.1080/00220388.2018.1448069

**Published:** 2018-03-20

**Authors:** Lu Gram, Joanna Morrison, Naomi Saville, Shyam Sundar Yadav, Bhim Shrestha, Dharma Manandhar, Anthony Costello, Jolene Skordis-Worrall

**Affiliations:** * Institute for Global Health, University College London, London, UK; ** Mother Infant Research Activities, Kathmandu, Nepal; † Department of Maternal, Newborn, Child and Adolescent Health, World Health Organization, Geneva, Switzerland

## Abstract

Participatory learning and action women’s groups (PLA) have proven effective in reducing neonatal mortality in rural, high-mortality settings, but their impacts on women’s agency in the household remain unknown. Cash transfer programmes have also long targeted female beneficiaries in the belief that this empowers women. Drawing on data from 1309 pregnant women in a four-arm cluster-randomised controlled trial in Nepal, we found little evidence for an impact of PLA alone or combined with unconditional food or cash transfers on women’s agency in the household. Caution is advised before assuming PLA women’s groups alone or with resource transfers necessarily empower women.

## 1. Introduction

Women’s groups practising participatory learning and action (PLA) are increasingly endorsed as a policy intervention to improving maternal and child health and empowering women worldwide (Victora & Barros, 2013). The World Health Organization explicitly recommends the implementation and scale-up of women’s groups practising PLA to improve newborn mortality and health (World Health Organization, 2014) and the Global Strategy for Women’s, Children’s and Adolescent’s Health for 2016–2030 promotes women’s groups as a strategy for engaging communities in health (Every Women Every Child, 2017).

The PLA approach is inspired by the philosophy of social activist Paulo Freire who advocated for engaging socially marginalised communities in collective action to tackle the societal roots of poverty and exclusion (Freire, 1972). With the help of a facilitator, group members are led through a PLA cycle to plan and implement strategies to address local health problems (Morrison et al., 2010). A meta-analysis of women’s group trials practising PLA in 2013 found an overall 20 per cent reduction in neonatal mortality (OR 0.80, 95% CI 0.67–0.96) and a non-significant 23 per cent reduction in maternal mortality (0.77, 0.48–1.23) in intervention areas when compared with control areas. A sub-group analysis found in trials with at least 30 per cent of pregnant women participating, that the intervention led to a 49 per cent reduction in maternal mortality (0.51, 0.29–0.89) and a 33 per cent reduction in neonatal mortality (0.67, 0.60–0.75) (Prost et al., 2013). One of the most frequently cited benefits of women’s groups practising PLA is their assumed ability to empower women (Prost et al., 2013; Rosato et al., 2008; Victora & Barros, 2013), but little evidence exists on their impact on women’s agency in the household.

Unconditional or conditional cash and food transfers have also become a popular policy tool for achieving a diverse range of outcomes including social protection (Holmes & Uphadya, 2009) and improvements in health, education and nutrition indicators (Adato & Hoddinott, 2010; Murray, Hunter, Bisht, Ensor, & Bick, 2014). Policies of cash and food transfer programmes usually target female beneficiaries because theoretical and empirical literature suggests that women are more likely to use resources on family and child wellbeing than men (Yoong, Rabinovich, & Diepeveen, 2012) and because such transfers may strengthen women’s bargaining power (Yoong et al., 2012). Yet, despite decades of transfer programmes targeting female beneficiaries, the existing body of evidence is still inconclusive on the effects of cash transfers on women’s family power (Bastagli et al., 2016). Evidence indicates an overall trend towards reductions in physical and sexual abuse, while some studies report increased emotional abuse and controlling behaviour (Bastagli et al., 2016). Evidence for impact on decision-making power is highly mixed (Bastagli et al., 2016).

The Low Birth Weight South Asia Trial (LBW-SAT) was a cluster-randomised controlled trial evaluating the impact on birth weight of women’s groups practising PLA either alone or combined with unconditional cash or food transfers (Saville et al., 2016). Low birth weight is a risk factor for lifelong health problems that affects 20 million newborns annually (Qadir & Bhutta, 2009) and underlies 80 per cent of neonatal deaths in sub-Saharan Africa and South Asia (Lawn et al., 2014). In many parts of South Asia, married women are expected to eat last and least in the family (Acharya & Bennett, 1983; Zaidi, 1996). While experiencing restricted mobility outside the home due to social norms of female propriety and modesty, they are also expected to defer to their husband and in-laws in matters of decision-making inside the home (Acharya & Bennett, 1983; Mandelbaum, 1993). Low decision-making power is in turn associated with poor maternal nutrition (Malapit, Kadiyala, Quisumbing, Cunningham, & Tyagi, 2013) and low birth weight (Chakraborty & Anderson, 2011). Qualitative studies in Bangladesh and Nepal have found that women overwhelmingly cite discriminatory food allocation and low decision-making power as barriers to dietary intake during pregnancy (Shannon, Mahmud, Asfia, & Ali, 2008; Morrison et al., 2018). Thus, as well as being of intrinsic value, lack of agency in the household may be a key barrier to increasing birth weight.

In this study, we report on the first quantitative randomised controlled evaluation of a PLA women’s group intervention, with and without unconditional food and cash transfers, on women’s agency within the household. Our study uses a measurement tool for capturing women’s agency proposed initially by the Oxford Poverty and Human Development Initiative (Ibrahim & Alkire, 2007) and adapted and validated by the study team for use in this trial (Gram et al., 2016).

## 2. Intervention design

This study was nested within a four-arm cluster-randomised trial comparing: 1) women’s groups practising PLA; 2) PLA women’s groups combined with unconditional cash transfers; and 3) PLA women’s groups combined with unconditional food transfers; against 4) a control arm. The unit of randomisation was the Village Development Committee (VDC), a sub-district level geographical area with approximate populations of 6000 each.^1^ Eighty VDCs were randomised to one of the four above treatments resulting in 20 VDCs per trial arm. Birth weight was the primary trial outcome. Compared to the control arm, mean birthweight was significantly higher in the PLA plus food arm, but not in the PLA only or PLA plus cash arms (Saville et al., in press). Trial interventions ran from 13 February 2014 until 10 October 2015. The full design of the trial has been reported elsewhere (Saville et al., 2016).

Government-paid female community health volunteers (FCHV) ran perinatal women’s groups prior to the introduction of the trial, but these groups met irregularly and had no specific agenda. These groups became the platform for our behaviour change strategy based on participatory learning and action. FCHVs and a hired assistant facilitator convened women’s groups where facilitators and group attendees discussed issues related to maternal nutrition and low birthweight. No meeting was explicitly devoted to women’s power and agency, but facilitators were open to discussing it, if raised by group members themselves. Groups voted on and implemented strategies to address prioritised problems, which were identified over a series of meetings. At the end of strategy implementation, the groups evaluated their efforts and began prioritising new health problems to tackle.

In the combined PLA and cash or food arms, meetings would end with an additional discussion on how to make best use of the transfer before it was distributed.^2^ Pregnant women were eligible for monthly transfers from the third month of pregnancy until the end of pregnancy. Women were not required to attend entire meetings to receive their transfers although they were encouraged to do so. Women who were physically unable to attend could receive transfers through home visits. The monthly cash transfer of NPR 750 (USD 7.3 equivalent as of September 2017) was designed to cover the cost of micronutrient-rich items such as dairy, meat, fish, eggs and fruits, but not a full basket of staple foods. The monthly food transfer consisted of 10 kg of Super Cereal, produced by World Food Program (UNICEF, 2014).

## 3. Conceptual framework

Despite extensive debate and reconceptualisation of the ‘empowerment’ concept in international development over the last 40 years, no single, clear definition has emerged (Trommlerová, Klasen, & Lessmann, 2015). One approach follows the suggestion by Ibrahim and Alkire (Ibrahim & Alkire, 2007) to view empowerment as an expansion of ‘agency freedom’, or just ‘agency’, an construct integral to the Capability Approach founded by Amartya Sen (1985). Out of multiple possible interpretations of the ‘agency’ construct itself, (Gram et al., 2016), we specifically define agency in this paper to refer to the degree to which an individual’s actions are in line with their own values, goals and interests in the face of support or opposition from their family members.

No single canonical theory exists of how women’s groups practising PLA achieve their health impacts either. This is especially so for mechanisms involving women’s agency in the household, as existing qualitative studies have overwhelmingly focused on community empowerment to the exclusion of agency in the household (Morrison, 2009; Rath et al., 2010; Rosato, 2012). We hypothesise three mechanisms through which women’s groups affect women’s agency in the household.

First, strong social norms prohibit the uninhibited physical movement of newly married women outside the household in our context (Davis, 2009). Physically leaving the confines of the home usually requires explicit permission from a senior family member and the provision of a suitable escort. Requests to go outside need to be phrased with a valid purpose and are not guaranteed to be approved. Local women and men use the colloquialism, ‘the frog in the well’ (*inaar ke beng*), to describe the sense of being trapped in the house. Thus, women who desire to socialise with others besides their immediate family members might find their agency enhanced by the intervention by making it easier for them to negotiate leaving the house, as the women’s groups specifically invited daughters-in-law to attend.

On the other hand, family members might begin to force pregnant women to attend groups who otherwise prefer to spend their time in other ways. Just like daughters-in-law need the permission of their elders to leave, so it is often impossible for them to refuse, if they are told to leave the house. However, passive participation due to family pressure diminishes agency just as much as non-participation due to family pressure. Researchers have heavily criticised mobility-based measures of empowerment for assuming that women desire mobility without asking them if this is the case (Mumtaz & Salway, 2005). Finally, it may be that only the women who are already able and willing to move about in public attend the meetings, while the remaining women stay at home. In this scenario, women’s agency would remain unchanged.

Second, the trial interventions aimed to promote healthy behaviours during pregnancy including adequate rest, health-seeking, and adequate dietary intake. Both women’s group facilitators and home visitors provided explicit messages to family members about the importance of these behaviours and women’s group strategies were oriented towards raising awareness about such messages in the community. In our context, such behaviours are often considered rare privileges for young daughters-in-law who are usually given the hardest and least desirable domestic tasks (Davis, 2003) and made to eat least and last in the family (Zaidi, 1996). In case of a felt need to visit the local health facility, daughters-in-law may also be denied the financial resources to do so (Simkhada, Porter, & van Teijlingen, 2010). Thus, daughters-in-law might find their agency enhanced by the ability to choose fewer or less arduous domestic tasks, by having a greater say over food in the household, or by being able to visit the healthcare facility whenever they themselves feel the need for it.

On the other hand, the trial interventions might only enable the few daughters-in-law who already have the support of their household to take up the recommended behaviours. Such a situation would improve our indicators on women’s actual health behaviours, while leaving measures of agency unaltered. Daughters-in-law might even experience diminished agency if family members begin to force them to follow NGO recommendations without ensuring daughters-in-law understand the rationale behind such recommendations.

Finally, in the best-case scenario, the many activities carried out by women’s group members and facilitators in the community to promote perinatal health might combine with an increased confidence on the part of pregnant women themselves to cause broader improvements to their position at home. Young daughters-in-law’s newfound status as a person who knows about community matters and participates in community meetings might subtly alter family members’ interactions with them. Their experience with problem-solving and planning within the PLA women’s groups is thought to engender greater confidence to negotiate, solve problems and plan for their own pregnancy at home. Women’s wider bargaining power is also thought to improve due to the presence of social support from women’s group members. The receipt of cash or food transfers is further hypothesised to strengthen their overall bargaining power by providing an independent source of income (Doss, 2013).

Improvements in women’s overall position at home might affect domains of life that are not directly related to the health promotion activities of the trial interventions. For example, if we saw an increase in women’s experience of agency in work outside the home, this might indicate a broader transformation of their opportunities.

## 4. Methods

### 4.1. Study context

The study took place in the Dhanusha and Mahottari districts of Nepal. With a GDP per capita of PPP USD 2573, Nepal is classed as a low-income country. Twenty-nine per cent of GDP comes from foreign remittances and 70 per cent of the population depends on agriculture for its livelihood (CIA, 2016). The total population of the two study districts is 1.4 million, 87 per cent are Hindu and 11 per cent Muslim. The districts are situated in the Plains belt of Nepal, an ecological zone with cultural similarities to areas of North India, Bangladesh and Pakistan. The districts are also the top two districts in Nepal for out-migration of domestic labour to foreign countries (Ministry of Labour and Migration, 2014). Apart from India, with whom Nepal shares open borders, top destinations for economic migrants are Qatar, Kuwait, United Arab Emirates and Malaysia (Ministry of Labour and Migration, 2014). Ninety-four per cent of migrant workers are men (Ministry of Labour and Migration, 2014). The local language and majority culture is Maithili. Married women live with their husband’s families in extended family groups and are culturally expected to defer to their husband and in-laws in decision-making matters.^3^ Twenty-one per cent of households in the region are classified as severely food insecure (Ministry of Health and Population (MOHP) [Nepal], New ERA, & ICF International Inc., 2012).

### 4.2. Measuring agency

Deci and Ryan proposed a survey tool for measuring self-determination in behaviour called the Relative Autonomy Index (Deci & Ryan, 2000). Alkire (Alkire, 2005) proposed this index as a measure of women’s agency due to its closeness of fit to Amartya Sen’s notion of agency (Sen, 1985). Our *primary measure of agency* was adapted from the tool used in Chad (Vaz, Pratley, & Alkire, 2016). Details about the adaptation and psychometric validation of the tool for our specific context have been reported elsewhere (Gram et al., 2016). *Secondary measures of agency* used for sensitivity testing included decision-making in large household purchases, food preparation and serving, and women’s own pregnancy, perceived ability to be an agent in one’s life (Ibrahim & Alkire, 2007) and perceived position on a ‘Power Ladder’ (Lokshin & Ravallion, 2005). Details about index construction can be found in the Supplementary Materials.

### 4.3. Statistical methodology

Details about sampling, data collection and analyses of missing data are provided in Appendix A. For our primary measure of agency, we conducted bootstrap regression of each of our agency outcomes on trial intervention area adjusting for geographical clustering using re-sampling on clusters (StataCorp, 2017) and regressing on additional covariates in case of unexpected imbalances between trial arms and to improve the precision of our main estimates. For all secondary measures, we carried out an ordered logistic regression adjusting for clustering with Huber robust estimators, using the same controls as in our main outcome analysis.

We adjusted for the following covariates: current maternal age and education, respondent’s age at marriage, head of household, separated/joint family, migrant labour of husband, residence in husband versus maternal home, caste, total number of children alive, number of sons currently alive, socio-economic status and spousal age and educational differences. These covariates were selected based on prior anthropological evidence from our context predicting the importance of age, fertility, family structure and caste (Acharya & Bennett, 1983; Cameron, 1995; Davis, 2009), and quantitative studies of the relationship between education, socio-economic status and women’s empowerment (Acharya, Bell, van Tijlingen, & Regmi, 2010; Jejeebhoy, 1995). Male labour migration has also been hypothesised to be associated with decision-making power in the household in our context (Kaspar, 2006).

Previous work on the Relative Autonomy Index also showed potentially high levels of inter-rater variability in our context (Gram et al., 2016). To control for systematic bias, we added fixed interviewer dummies. Each interviewer was allocated to multiple clusters spread across intervention and control areas, so multicollinearity was not an issue. The variance inflation factors were <2.6 for all interviewer dummies, where serious multicollinearity is usually only indicated for variance inflation factors above five (O’brien, 2007).

## 5. Results

### 5.1. Descriptive statistics

Overall, differences between trial arms in education, age, religion, caste, ethnicity, family structure, work, finances, and wealth were negligible (Table 1), although we observed some differences in spousal age and educational differences; in the control arm, husbands were on average 4.47 years older than their wives and had 1.35 more years of education, while husbands were on average 5.32–5.51 years older and had 1.70–2.59 more years of education than their wives in the intervention arms (p < 0.01 for a difference between arms).

**Table 1.** Descriptive statistics (n = 1309)

	PLA only	PLA and cash	PLA and food	Control	Overall	p-value
**Religion, caste and ethnicity**						
Hindu	0.82 (0.04)	0.83 (0.03)	0.84 (0.05)	0.80 (0.04)	0.83 (0.02)	0.94
Muslim	0.18 (0.04)	0.17 (0.03)	0.16 (0.05)	0.20 (0.04)	0.17 (0.02)	0.30
Dalit	0.17 (0.03)	0.13 (0.02)	0.13 (0.03)	0.19 (0.03)	0.15 (0.01)	0.94
Janajati	0.09 (0.02)	0.04 (0.02)	0.09 (0.03)	0.10 (0.03)	0.07 (0.01)	0.26
**Family structure**						
Head of household herself	0.11 (0.02)	0.10 (0.01)	0.09 (0.01)	0.10 (0.02)	0.10 (0.01)	0.85
Living separately from in-laws	0.10 (0.01)	0.09 (0.02)	0.10 (0.01)	0.08 (0.02)	0.09 (0.01)	0.86
No. of children alive	1.34 (0.07)	1.36 (0.08)	1.43 (0.07)	1.48 (0.12)	1.40 (0.04)	0.51
No. of sons currently alive	0.51 (0.04)	0.52 (0.03)	0.55 (0.04)	0.59 (0.04)	0.54 (0.02)	0.33
Husband migrated abroad	0.40 (0.03)	0.32 (0.03)	0.34 (0.03)	0.38 (0.03)	0.36 (0.01)	0.23
Woman residing in her husband’s home	0.98 (0.01)	0.96 (0.01)	0.96 (0.01)	0.99 (0.01)	0.97 (0.01)	0.13
**Age and education (years)**						
Woman’s current age (years)	23.2 (0.4)	23.3 (0.2)	23.6 (0.2)	23.9 (0.4)	23.5 (0.1)	0.40
Woman’s age at marriage	15.9 (0.2)	16.0 (0.1)	16.2 (0.2)	16.1 (0.2)	16.1 (0.1)	0.33
Husband age minus wife’s age	5.51 (0.29)	5.32 (0.22)	5.41 (0.26)	4.47 (0.22)	5.24 (0.13)	<0.01
Woman’s education (years)	2.35 (0.31)	2.84 (0.25)	2.23 (0.20)	2.42 (0.22)	2.50 (0.13)	0.12
Husband’s education minus wife’s education	2.33 (0.33)	1.70 (0.12)	2.59 (0.20)	1.35 (0.29)	2.02 (0.12)	<0.01
**Work, finances and wealth**						
Working outside the home	0.24 (0.04)	0.23 (0.02)	0.25 (0.02)	0.27 (0.02)	0.25 (0.01)	0.71
No. ‘domestic’ tasks^a^	3.45 (0.07)	3.21 (0.08)	3.47 (0.04)	3.39 (0.11)	3.36 (0.04)	0.11
No. ‘social’ tasks^a^	1.47 (0.13)	1.52 (0.08)	1.47 (0.09)	1.74 (0.12)	1.54 (0.05)	0.31
No. ‘outdoor’ tasks^a^	0.38 (0.04)	0.35 (0.03)	0.31 (0.04)	0.31 (0.04)	0.34 (0.02)	0.69
Having private savings	0.28 (0.02)	0.30 (0.03)	0.27 (0.03)	0.20 (0.04)	0.27 (0.01)	0.20
Wealth index (SD)	0.08 (0.11)	0.06 (0.06)	−0.07 (0.06)	−0.09 (0.09)	0.00 (0.04)	0.28
**Health and nutrition**						
Would seek care in a health facility for a health problem	0.51 (0.05)	0.49 (0.06)	0.61 (0.05)	0.47 (0.05)	0.53 (0.03)	0.24
Experienced food insecurity in the past year	0.13 (0.04)	0.13 (0.03)	0.10 (0.02)	0.12 (0.03)	0.12 (0.02)	0.91
**Community interaction**						
Any friend outside the home	0.58 (0.06)	0.64 (0.05)	0.59 (0.03)	0.63 (0.05)	0.61 (0.02)	0.78
Attending any groups	0.43 (0.03)	0.97 (0.01)	0.91 (0.02)	0.12 (0.03)	0.70 (0.04)	<0.01
Attending a non-trial group	0.15 (0.03)	0.07 (0.01)	0.10 (0.02)	0.12 (0.03)	0.10 (0.01)	0.08
**Engagement with the trial intervention**						
Heard of PLA women’s groups	0.52 (0.04)	0.97 (0.01)	0.98 (0.01)	0.05 (0.01)	0.72 (0.04)	<0.01
Participated in PLA women’s groups (1–3 times)	0.22 (0.03)	0.11 (0.02)	0.20 (0.02)	0.00 (0.00)	0.14 (0.01)	<0.01
Participated in PLA women’s groups (4+ times)	0.12 (0.02)	0.85 (0.03)	0.70 (0.03)	0.00 (0.00)	0.51 (0.04)	<0.01

*Notes*: ^a^Mean number of tasks are presented for the number of household tasks. For all other variables, mean proportions are shown. Robust standard errors are shown in brackets. ‘Domestic’ tasks comprised cooking, cleaning the house, performing small repairs, washing the dishes and doing laundry. ‘Social’ tasks comprised caring for elderly people, ill children and teaching children manners. ‘Outdoor’ tasks comprised shopping and managing the household money.

We also did not find evidence for a difference between trial arms in terms of covariates that we might conceivably expect the intervention to affect: uptake of employment outside the home, the allocation of domestic tasks at home, the tendency to seek healthcare, or the availability of friendships (Table 1). However, participation in women’s groups practising PLA differed substantially between intervention arms. Thirty-four per cent of women reported having ever participated in a women’s group in the PLA only arm, while 90–96 per cent reported having ever participated in the combined PLA and transfer arms. Rates of group participation in groups unrelated to the trial intervention were also low (10%), indicating the majority of pregnant women participating in groups of any kind only attended the women’s groups practising PLA.

### 5.2. Main trial impacts on agency

Descriptive statistics on primary and secondary outcomes are shown in Table 2.^4^ Table 3 shows the main trial impact. Impact estimates were close to zero for all domains except group participation, where we found strong evidence for increased agency (p < 0.01). This corresponded to a standardised increase of 0.31 standard deviations (SD) for PLA only, 0.79 SD for PLA and cash and 0.68 SD for PLA and food (not shown in table). We also found raised levels of external motivation in group participation for the two transfer arms (0.26 SD, p = 0.03 for PLA and cash; 0.34 SD, p < 0.01 for PLA and food), but no evidence for this in the women’s group only arm (−0.15 SD, p = 0.21 for PLA only). With respect to internal motivation, most estimates were close to zero in domains unrelated to group participation. We found strong evidence for an impact on internal motivation in group participation in all three arms of the trial (0.24 SD, p < 0.01 for PLA only arm; 1.20 SD, p < 0.01 for PLA and cash; 1.14 SD, p < 0.01 for PLA and food). An interpretation of these findings is provided in Section 6.1.

**Table 2.** Descriptive statistics of primary and secondary outcome measures (n = 1309)

	PLA only	PLA and cash	PLA and food	Control	Overall
**Work outside the home**					
Agency freedom	−0.51 (0.18)	−0.50 (0.13)	−0.85 (0.13)	−0.73 (0.14)	−0.64 (0.07)
External motivation	2.63 (0.10)	2.84 (0.08)	3.01 (0.07)	2.83 (0.11)	2.85 (0.05)
Internal motivation	2.13 (0.14)	2.34 (0.11)	2.16 (0.11)	2.11 (0.10)	2.21 (0.06)
**Household chores**					
Agency freedom	0.92 (0.11)	1.02 (0.09)	0.68 (0.08)	0.89 (0.10)	0.88 (0.05)
External motivation	2.65 (0.12)	2.76 (0.09)	3.00 (0.10)	2.71 (0.12)	2.80 (0.05)
Internal motivation	3.57 (0.08)	3.77 (0.06)	3.68 (0.07)	3.60 (0.09)	3.68 (0.04)
**Health-seeking**					
Agency freedom	0.45 (0.11)	0.60 (0.12)	0.33 (0.10)	0.68 (0.19)	0.51 (0.07)
External motivation	2.98 (0.12)	3.08 (0.13)	3.40 (0.12)	2.87 (0.17)	3.11 (0.07)
Internal motivation	3.43 (0.08)	3.68 (0.06)	3.72 (0.05)	3.55 (0.07)	3.62 (0.03)
**Group participation**					
Agency freedom	0.02 (0.18)	0.86 (0.15)	0.42 (0.10)	−0.36 (0.17)	0.35 (0.09)
External motivation	2.24 (0.14)	2.75 (0.14)	3.11 (0.11)	2.31 (0.15)	2.67 (0.08)
Internal motivation	2.25 (0.09)	3.61 (0.09)	3.53 (0.08)	1.94 (0.08)	3.02 (0.09)
**Large purchases**					
Sole decision-maker	0.08 (0.01)	0.05 (0.02)	0.05 (0.01)	0.07 (0.02)	0.06 (0.01)
Joint decision-maker	0.26 (0.04)	0.25 (0.03)	0.23 (0.04)	0.25 (0.02)	0.25 (0.02)
Not involved, but able to	0.26 (0.03)	0.41 (0.04)	0.33 (0.03)	0.28 (0.04)	0.34 (0.02)
Not involved and unable to	0.40 (0.04)	0.28 (0.04)	0.38 (0.04)	0.40 (0.04)	0.36 (0.02)
**Food preparation and serving**					
Sole decision-maker	0.56 (0.04)	0.52 (0.02)	0.56 (0.03)	0.56 (0.04)	0.55 (0.02)
Joint decision-maker	0.07 (0.02)	0.07 (0.02)	0.05 (0.01)	0.09 (0.02)	0.07 (0.01)
Not involved, but able to	0.23 (0.04)	0.33 (0.03)	0.25 (0.03)	0.23 (0.04)	0.27 (0.02)
Not involved and unable to	0.14 (0.03)	0.07 (0.02)	0.14 (0.02)	0.12 (0.03)	0.11 (0.01)
**Own pregnancy**					
Sole decision-maker	0.17 (0.03)	0.16 (0.03)	0.14 (0.02)	0.19 (0.04)	0.16 (0.01)
Joint decision-maker	0.27 (0.04)	0.29 (0.04)	0.25 (0.04)	0.29 (0.04)	0.28 (0.02)
Not involved, but able to	0.27 (0.03)	0.42 (0.03)	0.41 (0.03)	0.33 (0.04)	0.37 (0.02)
Not involved and unable to	0.29 (0.05)	0.14 (0.03)	0.20 (0.02)	0.19 (0.05)	0.19 (0.02)
**Step on the Power Ladder**	4.55 (0.31)	5.27 (0.17)	4.68 (0.18)	4.72 (0.17)	4.86 (0.11)
**Main agent of change in own life**					
No change desired	0.30 (0.05)	0.19 (0.04)	0.19 (0.05)	0.17 (0.05)	0.21 (0.02)
Woman herself	0.29 (0.05)	0.28 (0.05)	0.26 (0.06)	0.33 (0.08)	0.29 (0.03)
Not the woman herself	0.42 (0.05)	0.52 (0.05)	0.55 (0.08)	0.50 (0.07)	0.51 (0.03)

*Notes*: PLA refers here to participatory learning and action with women’s groups. For agency freedom, external motivation, internal motivation and the Power Ladder question, mean scores are presented. For the remaining indicators, mean proportions are presented. Robust standard errors in brackets.

**Table 3.** Main trial impacts on agency (n = 1309)

	PLA only versus control	PLA and cash versus control	PLA and food versus control
**Work outside the home**			
Agency	−0.02	−0.01	−0.02
	(0.20)	(0.18)	(0.14)
External motivation	−0.16	−0.07	0.01
	(0.13)	(0.14)	(0.12)
Internal motivation	−0.17	−0.08	0.00
	(0.18)	(0.14)	(0.14)
**Household chores**			
Agency	0.09	0.20	−0.04
	(0.11)	(0.13)	(0.12)
External motivation	−0.02	−0.06	0.10
	(0.11)	(0.13)	(0.11)
Internal motivation	0.07	0.15*	0.05
	(0.08)	(0.08)	(0.08)
**Health-seeking**			
Agency	−0.05	0.00	−0.07
	(0.13)	(0.16)	(0.12)
External motivation	0.00	0.04	0.14
	(0.10)	(0.13)	(0.09)
Internal motivation	−0.05	0.03	0.07
	(0.08)	(0.08)	(0.07)
**Group participation**			
Agency	0.53***	1.33***	1.14***
	(0.18)	(0.19)	(0.17)
External motivation	−0.19	0.34**	0.44***
	(0.15)	(0.15)	(0.14)
Internal motivation	0.33**	1.67***	1.58***
	(0.14)	(0.11)	(0.11)

*Notes*: PLA refers here to participatory learning and action with women’s groups. Mean effects with robust standard errors provided in brackets. Agency scores range from −4 to +4. Internal and external motivation scores range from 0 to 4. Control covariates are listed in Section 4.3. *Significant at 10 per cent; **significant at 5 per cent; ***significant at 1 per cent.

### 5.3. Secondary outcomes

Table 4 shows the estimated trial impacts on secondary outcomes of the trial. We did not find evidence for an impact on household decision-making over either large household purchases, food preparation and serving, or women’s own pregnancy. We also did not find evidence for an impact on the Power Ladder Question or the question on who was the main agent of change in women’s life. Indeed, in response to an open-ended prompt on what women wanted to change in their lives, only 3 per cent mentioned better maternal health (not shown in table). Fourteen per cent stated better education for themselves or their children, 22 per cent wanted jobs for themselves, 28 per cent stated acquisition of new land and property, and 21 per cent indicated that they did not desire change in their lives at this point. Among the women who desired a change, 35 per cent included themselves as one of the main agents of change, while 83 per cent named their husband, 48 per cent their in-laws, and 4 per cent their natal family. An interpretation of these findings is provided in Section 6.1.

**Table 4.** Trial impacts on secondary measures of agency (n = 1309)

	PLA only versus control	PLA and cash versus control	PLA and food versus control
**Household decision-making**			
Large household purchases	0.07	0.32	−0.06
	(0.21)	(0.23)	(0.21)
Food preparation and serving	−0.09	0.18	−0.09
	(0.21)	(0.18)	(0.18)
Own pregnancy	−0.08	0.25	−0.13
	(0.19)	(0.21)	(0.21)
**Power Ladder**	0.08	0.21	−0.06
	(0.20)	(0.15)	(0.14)
**Main agent of change in own life**	−0.14	−0.14	−0.15
	(0.17)	(0.17)	(0.20)

*Notes*: Mean log-odds ratios with robust standard errors in brackets are shown. The same controls are applied as in Table 4. *Significant at 10 per cent; **significant at 5 per cent; ***significant at 1 per cent.

## 6. Discussion

### 6.1. Interpretation of findings

Our results revealed little evidence for an impact on women’s agency in work outside the home, domestic work or health-seeking. Only agency in group participation was affected. Nor did we find evidence for an impact on women’s actual health-seeking behaviour or the actual allocation of domestic work. Nor did we find evidence for an impact on secondary measures of agency, such as measures of household decision-making, the Power Ladder Question or the question on who is the main agent of change in women’s lives. This indicates that the trial interventions neither had a broader impact on women’s bargaining power in the family, nor did they impact on agency in health behaviours.

In comparison to changes in women’s agency with greater age (Table 5), the impacts of the trial interventions on women’s agency seemed quite limited. With greater age, we found substantial increases in agency across all four domains, as well as increases in all secondary measures, as predicted by anthropological evidence (Acharya & Bennett, 1983; Davis, 2009). Such results also showed our measures were sensitive enough to detect broad changes in agency.

**Table 5.** Impact of age on agency (n = 1309)

	Main agency index				Decision-making				
Age of woman	Work outside the home	Household chores	Health-seeking	Group participation	Large purchases	Food serving & preparation	Own pregnancy	Power Ladder	Main agent of change in own life
<20 years	-	-	-	-	-	-	-	-	-
20–24 years	0.25*	0.15	−0.10	0.15	0.51***	0.35**	0.38**	0.37**	0.11
	(0.14)	(0.10)	(0.10)	(0.13)	(0.18)	(0.17)	(0.17)	(0.17)	(0.17)
25–29 years	0.63***	0.36**	0.11	0.55***	0.81***	0.71***	0.81***	0.42**	−0.02
	(0.19)	(0.15)	(0.11)	(0.18)	(0.24)	(0.23)	(0.21)	(0.20)	(0.23)
30–34 years	0.94***	0.37*	0.06	0.48**	1.03***	0.49	1.23***	0.27	0.38
	(0.28)	(0.20)	(0.17)	(0.21)	(0.27)	(0.34)	(0.29)	(0.31)	(0.31)
35+ years	1.44***	1.18***	0.40*	0.81***	1.41***	0.69	1.08***	0.84*	0.54
	(0.34)	(0.29)	(0.22)	(0.30)	(0.49)	(0.64)	(0.40)	(0.43)	(0.42)

*Notes*: The same covariates are adjusted for in the model as in Table 4. Mean effects are shown for the main agency index. Log-odds ratios are shown for decision-making, Power Ladder, and the question on the main agent of change in respondents’ own life. Robust standard errors provided in brackets. *Significant at 10 per cent; **significant at 5 per cent; ***significant at 1 per cent.

Its important to consider why our intervention might not have had broader impacts on women’s agency. Process evaluation of the operation of the women’s groups suggested these groups might not have been as effective at engendering community action as they were at getting women to attend group meetings (Morrison, Saville, Gram, & Harris-Fry, 2016). Women’s group facilitators reported that meetings to formulate and implement strategies were the most difficult to run. Indeed, one of the ‘strategies’ implemented by some women’s groups included home visits by women’s group facilitators, which made it unclear to what extent local women had ownership over the strategy implementation process in those groups. In general, groups overwhelmingly decided to implement extra community meetings to raise awareness about the importance of maternal nutrition. However, these meetings were challenging, because local norms made it unacceptable for women to speak in a public meeting and women themselves had little prior experience with public speaking. A few groups held separate meetings with men, but these tended to be ad hoc and few in number. In the group meetings themselves, it was nearly impossible to openly discuss the powerful role of mothers-in-law in deciding over the welfare of their daughters-in-law. Mothers-in-law often escorted their daughters-in-law to group meetings and closely watched the behaviour of their daughters-in-law over the course of the meeting.

This contrasts with past experience of participatory women’s groups in maternal and child health, which saw a wide diversity of strategies implemented by group members requiring considerable levels of sophistication, coordination and participation (Morrison, 2009; Rath et al., 2010; Rosato, 2012). Differences in context may have played a role. Maithili culture is known to have more restrictive gender norms about women’s role in public than the Hills of Nepal or the tribal areas of Jharkhand in India (Acharya & Bennett, 1983; Bennett, 1992), where previous women’s groups trials took place (Morrison et al., 2010; Rath et al., 2010). The introduction of material transfers may also have corrupted the participatory process, as group members became focused on the optimal use of incentives rather than community action in the PLA and transfer arms (Morrison et al., 2016). Indeed, women’s group facilitators reported that participants in the transfer arms of the intervention were particularly difficult to motivate to take lead roles in engaging their communities (Morrison et al., 2016). At the same time, group members in the PLA only arms might have become discouraged by learning from friends and relatives that women in other parts of their district received monthly resource transfers, while they only received a single lump sum (NPR 1000 [9.5 USD]) at the end of their pregnancy as compensation for participating in the trial (Saville et al., 2016). Finally, the emphasis of previous trials on reducing mortality may have served as a stronger focal point for community action than an objective of increasing birth weight.

In the absence of a strong, coordinated community response to gender issues in the household, the trial interventions may not have been able to create wider change in household norms. Young daughters-in-law likely needed considerable amounts of social support to alter their position in the household in our context, even with the aid of resource transfers. In turn, the lack of wider change in the position of daughters-in-law at home may have left them unable to demand changes to health-seeking behaviours or changes to their domestic work burden.

The observed impacts of all three intervention arms on women’s agency in the group participation domain relative to control did provide evidence for a small degree of agency enhancement in our trial. The effect sizes (0.53–1.14, Table 4) were meaningfully large, since these were comparable to the impacts of substantial changes in women’s age (0.81 for 35+ years compared to <18 years, Table 5). The most straightforward interpretation would be that usually cloistered daughters-in-law genuinely enjoyed getting the opportunity to leave the home and attend group meetings. For example, 91–98 per cent of daughters-in-law who participated in women’s groups in the intervention arms agreed with the statement ‘you participate, because you want to’, but only 37–52 per cent of daughters-in-law who did not participate agreed with the statement ‘you did not participate, because you did not want to’.

This was not merely an effect of the incentives, since we observed the impact in both incentivised and un-incentivised arms of the trial. Existing psychological evidence on the effect of incentives on intrinsic motivation suggests either no effect or a reduction in autonomy (Deci, Koestner, & Ryan, 1999; Promberger & Marteau, 2013), unless the incentives are interpreted by recipients themselves as respecting their autonomy (Eisenberger, Pierce, & Cameron, 1999). We also ruled out social desirability, since local social norms actively encourage a lack of agency in the newly married in our local context. Indeed, we found evidence for an increase in external motivation in group participation in the transfer arms, which was absent in the non-incentivised arm. This suggests that daughters-in-law felt free to report mixed motives regarding group participation in our trial.

Thus, although we cannot claim any large impacts on maternal agency in this trial, it is still important to respect local women’s own stated views about what is important and valuable to them by reporting their experienced increase in agency following participation in the women’s groups in this study.

### 6.2. Study limitations

We should emphasise that our tool primarily measured agency in family relations. Although process evaluation reports suggested the trial interventions did not have a strong impact on community empowerment (Morrison et al., 2016), we cannot draw such conclusions directly from our own data. It is also possible that broader changes in women’s agency might emerge after a longer time period than the span of the trial intervention (three years). It is also possible that women’s agency in domains that we did not measure were affected by the trial, for example, agency in child-care. Short-term subtle changes in household relationships and delicate changes in respondents’ experienced intensity of internal or external motivation may also have not been detected by our tool.

## 7. Conclusion

Our study is the first to evaluate the impact on women’s agency of women’s groups practising PLA as well as PLA with cash or food transfers. We found little evidence of broader impacts of women’s groups on domains such as work outside the household, household chores and health-seeking, although we found a limited increase in women’s agency in the group participation domain. Our lack of broad impact is surprising given the frequent claim that groups practising PLA ‘empower’ women (Rosato et al., 2008). Successful community mobilisation may be more challenging than anticipated and should not be taken for granted by development researchers and practitioners. Future research should continue to monitor the impact of women’s groups practising PLA on women’s empowerment in other contexts.

## Figures and Tables

**Figure A1.**
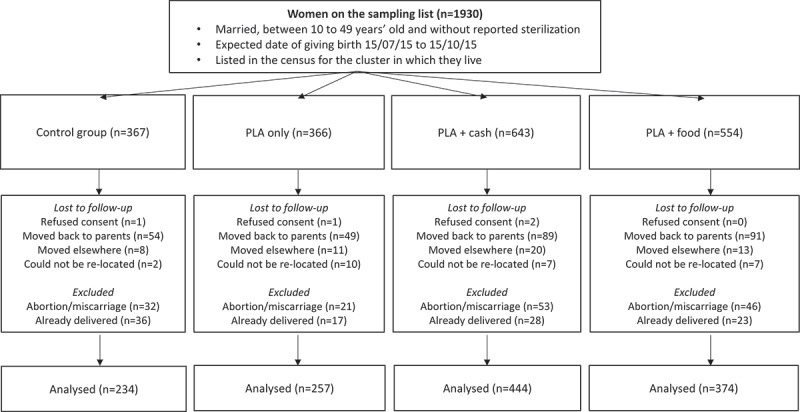
Sampling strategy and trial flow.

## Notes

1. The cluster randomised controlled design of the trial was specifically adopted to control for potential contamination between different individuals in the same geographical area. Since entire VDCs were randomised to one of the four treatment arms of the trial, we can rule out contamination between individuals living within the same VDC. The VDCs were chosen to be spaced sufficiently far apart to reduce the risk of contamination between VDCs to a minimum.

2. In the spirit of PLA, both women’s group members themselves and women’s group facilitators were supposed to be involved in defining the ‘best use’ of cash and food transfers in these discussions. However, given the significant disparity in formal status between facilitators and women’s group members, this was not always easy to achieve. A forthcoming paper is being prepared on this complex topic.

3. When husbands have migrated abroad, married women are still expected to defer to their husband on decision-making matters over the phone. Lu Gram observed a married woman being severely castigated by her husband over the phone after greeting our interviewers. Household members had phoned him to tell him that she had disobeyed his orders to speak to strangers without his explicit permission. Furthermore, in-laws hold even greater authority over married women than husbands in the Maithili family, and it is exceedingly rare for newly married Maithili women to live apart from their in-laws.

4. Note, there was considerable diversity in decision-making power between joint and separated households. For example, in 62% of households where the respondent lived together with her mother-in-law, the nuclear couple was completely excluded from decisions on large purchases. Among respondents living separately from their mother-in-law, the dominant pattern was exclusive decision-making on large purchases within the nuclear couple (84%). However, we saw no evidence for a difference in women living jointly or separately from their in-laws between trial arms (p = 0.86, see Table 1).

## Appendix

### Appendix A

#### A.1. Sampling and data collection

A household census conducted in 2013 before the start of the intervention identified married women between 10 and 49 years of age who had not had operative family planning and whose husbands had not had a vasectomy. These were asked to consent to take part in the trial surveillance system which identified new pregnancies over the course of the trial, a ‘menstrual monitoring system’ (Style et al., 2017). As discussed in Section 2, in all intervention areas, women’s groups practising PLA were open to all community members to attend, but cash and food transfers were only provided to consenting pregnant women from the third month of pregnancy until the end of pregnancy. Between 2013 and 2015, more than 60,000 women were enrolled into menstrual monitoring and more than 25,000 pregnancies were detected. The details of the trial surveillance system have been reported elsewhere (Style et al., 2017).

To evaluate the impact of the intervention on women’s agency for women at immediate risk of delivering low birth weight babies, we sub-sampled women from the total pool of enrolled women who were 7.00–7.99 months’ pregnant at any time between 15 June and 15 August 2015. We achieved this range of gestational ages by targeting women for interview with an expected date of delivery between the 15 July and 15 October and planning field work so that women were visited between one and two months before their delivery date. However, some women ended their pregnancy earlier than expected due to premature delivery, abortions or miscarriages. Women who were no longer pregnant by the time they were reached by the field worker were excluded.

We experienced some in-migration into the cash and food transfer arms of the trial from both within and outside the study area as women sought to become eligible for the transfers.^5^ In the presence of bias from more empowered women self-selecting into the transfer arms, we would expect to see an exaggerated intervention effect in the transfer arms. To reduce potential bias, we only included women listed on the aforementioned household census in 2013 before the start of the intervention.

After applying our inclusion and exclusion criteria to all pregnant women enrolled in the trial, we obtained 1930 women for our sampling list.

Figure A1 shows a CONSORT diagram for the sample selection process (Schulz, Altman, & Moher, 2010). In total, data were available for 1309 pregnant women. 152 cases were excluded due to miscarriage/abortion and 104 were excluded as they had already delivered a live or still birth by the time we reached them. 284 cases had temporarily migrated back to their maternal home, while 52 had moved elsewhere and 26 could not be located. The loss to follow-up rates were 22 per cent in the control arm, 22 per cent in the PLA only arm, 21 per cent in the PLA and cash arm and 23 per cent in the PLA and food arm. Four women did not consent to be interviewed.

Data collectors were trained in negotiation techniques to gain privacy for the interview due to the sensitive nature of the questions. Parents-in-law were advised of the importance of allowing the field worker to be alone with the daughter-in-law for the purposes of our research. Eighty-eight per cent of the interviews were conducted alone with the respondent, 5 per cent were conducted in the presence of the mother-in-law only, 2 per cent in the presence of the husband only and 2 per cent in the presence of other women in the household only. The remaining 3 per cent were accounted for by various combinations of male and female household members as well as neighbours.

#### A.2. Handling missing data

##### A.2.1. Dealing with survey non-response

Out of 1930 women on our original sampling list, we interviewed 1309 on their agency. To check for bias due to loss of follow-up, we compared these women with women who were not interviewed in terms of socio-demographic indicators. We obtained this information on the 365 women who were unavailable for interview in our study by merging data from our study from a baseline survey administered to all enrolled women in LBWSAT and re-visiting those (n = 41) who had not been administered this baseline survey.

**Table A1.** Respondent characteristics for intervention and control arms, with (n = 365) and without (n = 1309) missing empowerment data

		**Included in main analysis (n = 1309)**				
	PLA only	PLA and cash	PLA and food	Control	Overall	p-value
Education (years)	2.35 (0.31)	2.84 (0.25)	2.23 (0.20)	2.42 (0.22)	2.50 (0.13)	0.12
Age (years)	23.2 (0.4)	23.3 (0.2)	23.6 (0.2)	23.9 (0.4)	23.5 (0.1)	0.40
Hindu	0.82 (0.04)	0.83 (0.03)	0.84 (0.05)	0.80 (0.04)	0.83 (0.02)	0.94
Muslim	0.18 (0.04)	0.17 (0.03)	0.16 (0.05)	0.20 (0.04)	0.17 (0.02)	0.30
Dalit	0.17 (0.03)	0.13 (0.02)	0.13 (0.03)	0.19 (0.03)	0.15 (0.01)	0.94
Janajati	0.09 (0.02)	0.04 (0.02)	0.09 (0.03)	0.10 (0.03)	0.07 (0.01)	0.26
No. of children alive	1.34 (0.07)	1.36 (0.08)	1.43 (0.07)	1.48 (0.12)	1.40 (0.04)	0.51
Wealth index (SD)	0.08 (0.11)	0.06 (0.06)	−0.07 (0.06)	−0.09 (0.09)	0.00 (0.04)	0.28
		**Lost to follow-up (n = 365)**				
	PLA only	PLA and cash	PLA and food	Control	Overall	p-value
Education (years)	3.42 (0.64)	3.87 (0.34)	2.71 (0.33)	3.19 (0.38)	3.30 (0.22)	0.14
Age (years)	21.9 (0.5)	21.4 (0.5)	22.0 (0.4)	22.3 (0.6)	21.8 (0.3)	0.73
Hindu	0.73 (0.08)	0.80 (0.05)	0.72 (0.06)	0.68 (0.07)	0.74 (0.03)	0.48
Muslim	0.27 (0.08)	0.20 (0.05)	0.27 (0.05)	0.32 (0.07)	0.26 (0.03)	0.69
Dalit	0.13 (0.04)	0.17 (0.04)	0.21 (0.05)	0.17 (0.06)	0.17 (0.02)	0.51
Janajati^a^	0.04 (0.02)	0.00 (0.00)	0.06 (0.03)	0.05 (0.03)	0.04 (0.01)	0.00
No. of children alive	0.99 (0.17)	0.97 (0.13)	1.16 (0.07)	0.89 (0.11)	1.02 (0.06)	0.10
Wealth index (SD)	−0.06 (0.16)	0.15 (0.10)	0.12 (0.09)	0.00 (0.11)	0.07 (0.06)	0.59

*Notes*: Means are provided with robust standard errors in brackets and global robust tests for differences across arms. Testing for differences between participants with and without missing data, education (p < 0.01), age (p < 0.01), Hindu religion (p < 0.01), Muslim religion (p < 0.01), Janajati ethnicity (p = 0.03) and number of children alive (p < 0.01) are strongly related to missingness. However, Dalit caste (p = 0.30) and wealth (p = 0.22) are not. ^a^Only 13 women lost to follow-up were Janajatis.

The differences between women who were interviewed on their agency and the women who were not are shown in Table A1. We found no evidence for a systematic difference in follow-up rates between trial arms (p = 0.83). The characteristics of women with missing data also did not vary across trial arms in ways dissimilar to women without missing data. Tests of interaction between intervention arm and missingness revealed no differences for all baseline characteristics (p > 0.3) except indigenous (Janajati) ethnicity (p < 0.01). However, the latter result was likely an artefact of the small number of Janajati women with missing data in total (n = 13).

However, women with missing data were different from women without missing data. These were on average younger, less likely to have a child, more educated, less likely to be Hindu (74 versus 83%) and more likely to be Muslim (26 versus 17%) than women without missing data. Women were also more likely to be of Janajati ethnicity (7 versus 4%), although no evidence for difference in Dalit caste and wealth were found (p > 0.2). As our adjusted analysis for the main effect already included a number of controls potentially associated with missingness and no additional variables in our dataset were plausibly associated with both missingness and agency, we did not perform additional analyses to control for survey non-response.

##### A.2.2 Dealing with item non-response

Forty-eight of the 1309 cases (3.6%) exhibited item non-response for at least one item in the main outcome data. Thirty-one had non-response to a single item, 12 to two items, three to three items, one to seven items and one to eight items. The total number of items in our battery was 32. As the level of item non-response was low, we performed single item imputation using a logistic regression model. In addition to the controls in our main analysis, we added female labour participation, private savings, food security, number of household chores performed by the respondent, group participation, decision-making on food and expenditures and the responses to all the other agency items with non-missing values. None of our conclusions changed if we instead dropped the women with item non-response. We did not have any cases with missing data in the control variables for our main analysis.

## References

[CIT0001] Acharya D. R., Bell J. S., van Tijlingen E. R., & Regmi P. R. (2010). Women’s autonomy in household decision-making: A demographic study in Nepal. *Reproductive Health*, 7, 1–12. 20630107 10.1186/1742-4755-7-15 PMC2914657

[CIT0002] Acharya M., & Bennett L. (1983). *Women and the subsistence sector. Economic participation and household decision-making in Nepal* (Rep. No. 526). Washington, DC: World Bank.

[CIT0003] Adato M., & Hoddinott J. (Eds.). (2010). Conditional cash transfers in the second decade: Current debates and new frontiers In *Conditional cash transfers in Latin America* (pp. 351–371). Washington, DC: International Food Policy Research Institute.

[CIT0004] Alkire S. (2005). Subjective quantitative studies of human agency. *Social Indicators Research*, 74, 217–260.

[CIT0005] Bastagli F., Hagen-Zanker J., Harman L., Barca V., Sturge G., & Schmidt T. (2016). *Cash transfers: What does the evidence say? A rigorous review of programme impact and the role of design and implementation features*. London: Overseas Development Institute.

[CIT0006] Bennett L. (1983). *Dangerous wives and sacred sisters* . New York: Columbia University Press.

[CIT0007] Bennett L. (1992). *Women, poverty and productivity in India* (Rep. No. 10595). Washington, DC: World Bank.

[CIT0008] Cameron M. M. (1995). Transformations of gender and caste divisions of labor in rural Nepal: Land, hierarchy, and the case of untouchable women. *Journal of Anthropological Research*, 51, 215–246.

[CIT0009] Chakraborty P., & Anderson A. K. (2011). Maternal autonomy and low birth weight in India. *Journal of Women’s Health*, 20, 1373–1382. 10.1089/jwh.2010.2428 21767141

[CIT0010] CIA (2016). *The World Factbook: Nepal. Central intelligence agency [Electronic version]*. Retrieved from https://www.cia.gov/AQ35library/publications/the-world-factbook/geos/np.html

[CIT0011] Davis C. (2003). Feminist tigers and patriarchal lions: Rhetorical strategies and instrument effects in the struggle for definition and control over development in Nepal. *Meridians*, 3, 204–249.

[CIT0012] Davis C. V. (2009). Im/possible lives: Gender, class, self-fashioning, and affinal solidarity in modern South Asia. *Social Identities*, 15, 243–272.

[CIT0013] Deci E. L., Koestner R., & Ryan R. M. (1999). A meta-analytic review of experiments examining the effects of extrinsic rewards on intrinsic motivation. *American Psychological Association*, 125, 627–668. 10.1037/0033-2909.125.6.627 10589297

[CIT0014] Deci E. L., & Ryan R. M. (2000). The” what” and” why” of goal pursuits: Human needs and the self-determination of behavior. *Psychological Inquiry*, 11, 227–268.

[CIT0015] Doss C. (2013). Intrahousehold bargaining and resource allocation in developing countries. *World Bank Research Observer*, 28, 52–78.

[CIT0016] Eisenberger R., Pierce W. D., & Cameron J. (1999). Effects of reward on intrinsic motivation - Negative, neutral, and positive: Comment on Deci, Koestner, and Ryan (1999). *Psychological Bulletin*, 125, 677–691. 10589299 10.1037/0033-2909.125.6.677

[CIT0017] Every Women Every Child (2017). *Global strategy for women’s children’s and adolescents’ health 2016-2030*. Retrieved from http://globalstrategy.everywomaneverychild.org/pdf/EWEC_globalstrategyreport_200915_FINAL_WEB.pdf

[CIT0018] Freire P. (1972). *Pedagogy of oppressed*. New York: Herder and Herder.

[CIT0019] Gram L., Morrison J., Sharma N., Shrestha B. P., Manandhar D. S., Costello A., ... Skordis-Worrall J. (2016). Validating an agency-based tool for measuring women's empowerment in a complex public health trial in rural Nepal. *Journal of Human Development and Capabilities*, 18(1), 107 –135. 28303173 10.1080/19452829.2016.1251403 PMC5327873

[CIT0020] Holmes R. E., & Uphadya S. (2009). *The role of cash transfers in post-conflict Nepal*. London: Overseas Development Institute.

[CIT0021] Ibrahim S., & Alkire S. (2007). Agency and empowerment: A proposal for internationally comparable indicators. *Oxford Development Studies*, 35, 379–403.

[CIT0022] Jejeebhoy S. J. (1995). *Women’s education, autonomy, and reproductive behaviour: Experience from developing countries*. Oxford: Clarendon Press.

[CIT0023] Kaspar H. (2006). I am the head of the household now: The impacts of outmigration for labour on gender hierarchies in Nepal (Part II) In Premchander S. & Muller C. (Eds.), *Gender and sustainable development: Case studies from NCCR North-South* (pp. 285–304). Bern: Geographica Bernensia.

[CIT0024] Lawn J. E., Blencowe H., Oza S., You D., Lee A. C. C., Waiswa P., … Cousens S. N. (2014). Every newborn: Progress, priorities, and potential beyond survival. *The Lancet*, 384, 189–205. 10.1016/S0140-6736(14)60496-7 24853593

[CIT0025] Lokshin M., & Ravallion M. (2005). Rich and powerful?: Subjective power and welfare in Russia. *Journal of Economic Behavior & Organization*, 56, 141–172.

[CIT0026] Malapit H. J., Kadiyala S., Quisumbing A. R., Cunningham K., & Tyagi P. (2013). *Women’s empowerment in agriculture, production diversity, and nutrition: Evidence from Nepal*. Washington, DC: International Food Policy Research Institute, Poverty Health and Nutrition Division.

[CIT0027] Mandelbaum D. G. (1993). *Women’s seclusion and men’s honor: Sex roles in North India, Bangladesh, and Pakistan*. Tucson: University of Arizona Press.

[CIT0028] Ministry of Health and Population (MOHP) [Nepal], New ERA, & ICF International Inc (2012). *Nepal demographic and health survey 2011*. Kathmandu: Ministry of Health and Population, New ERA and ICF International, Calverton, Maryland.

[CIT0029] Ministry of Labour and Migration (2014). *Labour migration for employment: A status report for Nepal 2013/2014* Kathmandu: Government of Nepal.

[CIT0030] Morrison J. (2009). *Understanding the effect of a participatory intervention with women’s groups to improve maternal and neonatal health in rural Nepal* (Thesis (Ph.D.)), UCL (University College London), London.

[CIT0031] Morrison J., Dulal S., Harris-Fry H., Basnet M., Sharma N., Shrestha B., ... Saville N (2018). Formative qualitative research to develop community-based interventions addressing low birth weight in the plains of Nepal. *Public Health Nutrition*, 21(2), 377 –384. 29032790 10.1017/S1368980017002646 PMC5756621

[CIT0032] Morrison, J., Saville, N., Gram, L., & Harris-Fry, H (2016). *A report of the Low Birth Weight South Asia Trial process evaluation exploring mechanisms of how interventions may have worked Kathmandu*. Nepal: University College London.

[CIT0033] Morrison J., Thapa R., Hartley S., Osrin D., Manandhar M., Tumbahangphe K., … Costello A. (2010). Understanding how women’s groups improve maternal and newborn health in Makwanpur, Nepal: A qualitative study. *International Health*, 2, 25–35. 24037047 10.1016/j.inhe.2009.11.004 PMC5104268

[CIT0034] Mumtaz Z., & Salway S. (2005). ‘I never go anywhere’: Extricating the links between women’s mobility and uptake of reproductive health services in Pakistan. *Social Science & Medicine*, 60, 1751–1765. 15686807 10.1016/j.socscimed.2004.08.019

[CIT0035] Murray S. F., Hunter B. M., Bisht R., Ensor T., & Bick D. (2014). Effects of demand-side financing on utilisation, experiences and outcomes of maternity care in low-and middle-income countries: A systematic review. *BMC Pregnancy and Childbirth*, 14, 1–15. 24438560 10.1186/1471-2393-14-30 PMC3897964

[CIT0036] O’brien R. M. (2007). A caution regarding rules of thumb for variance inflation factors. *Quality & Quantity*, 41, 673–690.

[CIT0037] Promberger M., & Marteau T. M. (2013). When do financial incentives reduce intrinsic motivation? Comparing behaviors studied in psychological and economic literatures. *Health Psychology*, 32, 950–957. 24001245 10.1037/a0032727 PMC3906839

[CIT0038] Prost A., Colbourn T., Seward N., Azad K., Coomarasamy A., Copas A., … Costello A. (2013). Women’s groups practising participatory learning and action to improve maternal and newborn health in low-resource settings: A systematic review and meta-analysis. *The Lancet*, 381, 1736–1746. 10.1016/S0140-6736(13)60685-6 PMC3797417 23683640

[CIT0039] Qadir M., & Bhutta Z. (2009). Low birth weight in developing countries In Kiess W, Chernausek S. D. & Hokken K. (Eds.), *Small for gestational age: Causes and consequences*. Leipzig: Pediatric and Adolescent Medicine.

[CIT0040] Rath S., Nair N., Tripathy P. K., Barnett S., Rath S., Mahapatra R., … Prost A. (2010). Explaining the impact of a women’s group led community mobilisation intervention on maternal and newborn health outcomes: The Ekjut trial process evaluation. *BMC International Health and Human Rights*, 10, 25. 20969787 10.1186/1472-698X-10-25 PMC2987759

[CIT0041] Rosato M. (2012). *How does community mobilisation through MaiMwana women’s groups work? Addressing the social determinants of mother and child health in rural Malawi* (Thesis (Ph.D.)), University College London, London.

[CIT0042] Rosato M., Laverack G., Grabman L. H., Tripathy P., Nair N., Mwansambo C., … Costello A. (2008). Community participation: Lessons for maternal, newborn, and child health. *The Lancet*, 372, 962–971. 10.1016/S0140-6736(08)61406-3 18790319

[CIT0043] Saville N. M., Shrestha B. P., Style S., Harris-Fry H., Beard B. J., Sen A., ... Costello A (in press). Impact on birthweight and child growth of Participatory Learning and Action women’s groups with and without transfers of food or cash during pregnancy: Findings of the Low Birth Weight South Asia cluster-randomised controlled trial (LBWSAT) in Nepal. *Plos One*. 10.1371/journal.pone.0194064 PMC5942768 29742136

[CIT0044] Saville N. M., Shrestha B. P., Style S., Harris-Fry H., Beard B. J., Sengupta A., ... Costello A (2016). Protocol of the low Birth Weight South Asia Trial LBWSAT), a cluster-randomised controlled trial testing impact on birth weight and infant nutrition of participatory learning and action through women's groups, with and without unconditional transfers of fortified food or cash during pregnancy in Nepal. *BMC Pregnancy and Childbirth*, 16, 1 –19. 27769191 10.1186/s12884-016-1102-x PMC5073870

[CIT0045] Schulz K. F., Altman D. G., & Moher D. (2010). CONSORT 2010 statement: Updated guidelines for reporting parallel group randomized trials. *Annals of Internal Medicine*, 152, 726–732. 20335313 10.7326/0003-4819-152-11-201006010-00232

[CIT0046] Sen A. (1985). Well-being, agency and freedom: The Dewey lectures 1984. *The Journal of Philosophy*, 82, 169–221.

[CIT0047] Shannon K., Mahmud Z., Asfia A., & Ali M. (2008). The social and environmental factors underlying maternal malnutrition in rural Bangladesh: Implications for reproductive health and nutrition programs. *Health Care for Women International*, 29, 826–840. 18726794 10.1080/07399330802269493

[CIT0048] Simkhada B., Porter M. A., & van Teijlingen E. R. (2010). The role of mothers-in-law in antenatal care decision-making in Nepal: A qualitative study. *BMC Pregnancy and Childbirth*, 10, 34. 20594340 10.1186/1471-2393-10-34 PMC2910658

[CIT0049] StataCorp L. P. (2017). *bootstrap - Bootstrap sampling and estimation* . The Stata Manual v.13 [Electronic version]. Retrieved from https://www.stata.com/manuals13/rbootstrap.pdf

[CIT0050] Style S., Beard B. J., Harris-Fry H., Sengupta A., Jha S., Shrestha B. P., ... Saville N. M (2017). Experiences in running a complex electronic data capture system using mobile phones in a large-scale population trial in Southern Nepal. *Global Health Action*, 10, 1330858. 28613121 10.1080/16549716.2017.1330858 PMC5496067

[CIT0051] The World Factbook: Nepal (2016). *Central intelligence agency [Electronic version]*. Retrieved from: https://www.cia.gov/library/publications/the-world-factbook/geos/np.html

[CIT0052] Trommlerová S. K., Klasen S., & Lessmann O. (2015). Determinants of empowerment in a capability-based poverty approach: Evidence from The Gambia. *World Development*, 66, 1–15.

[CIT0053] UNICEF (2014). *Technical bulletin no. 16: Supercereal products* (Rep. No. 2014). Copenhagen: Author.

[CIT0054] Vaz A., Pratley P., & Alkire S. (2016). Measuring women’s autonomy in chad using the relative autonomy index. *Feminist Economics*, 22, 264–294.

[CIT0055] Victora C. G., & Barros F. C. (2013). Participatory women’s groups: Ready for prime time? *The Lancet*, 381, 1693–1694. 10.1016/S0140-6736(13)61029-6 23683618

[CIT0056] World Health Organization (2014). *WHO recommendation on community mobilization through facilitated participatory learning and action cycles with women’s groups for maternal and newborn health*. Geneva: Author. 25165805

[CIT0057] Yoong J., Rabinovich L., & Diepeveen S. (2012). *The impact of economic resource transfers to women versus men: A systematic review*. London: University of London (London, EPPI-Centre).

[CIT0058] Zaidi S. A. (1996). Gender perspectives and quality of care in underdeveloped countries: Disease, gender and contextuality. *Social Science & Medicine*, 43, 721–730. 8870136 10.1016/0277-9536(96)00116-5

