# Supplementary material for: Do Participatory Learning and Action Women’s Groups Alone or Combined with Cash or Food Transfers Expand Women’s Agency in Rural Nepal?
Source: J Dev Stud. 2018 Mar 20;55(8):1670–86. doi: 10.1080/00220388.2018.1448069 (PMC6540743; doi:10.1080/00220388.2018.1448069)
Supplement: Supplementary Materials [file FJDS_A_1448069_SM9669.pdf]

This supplementary file describes how household agency was measured and scored in our study using both primary and secondary measures. It also describes how a wealth index was created. Finally, a web table (W1) is provided listing all the items in our main empowerment module.

Scoring and measuring agency

Our primary measurement tool focused on women's agency in the household within four separate domains:

- Work outside the household
- Performance of household chores
- Health-seeking for moderate health problems
- Group participation

Respondents were asked about their motivation for behaviours in each domain and scored on whether these motivations were external or internal. Respondents who did not engage in any activities in a particular domain were asked about their motivation for refraining from doing so. Respondents' score on their external motivation increased by +1 for every external motivation they had, while their score on internal motivation increased by +1 for every internal motivation they had. Respondents' agency freedom score was calculated as internal motivation minus external motivation. Item scores were summed across all items in a specific domain to obtain domain-specific scores on external motivation, internal motivation and agency freedom. The entire list of questions concerning agency along with a scoring scheme are presented in Table W1.

In each domain, we created an agency score by summing all items, ranging from -4 to +4. In each domain, equal weight was given to those who carried out activities in that domain and those who refrained from any activity in order to avoid privileging activity over inactivity. We disaggregated our agency scores into external and internal motivation scores by counting the number of items measuring respectively external or internal motivation to which the respondent had agreed to. These scores ranged from 0 to +4.

Three secondary measures of agency were also employed. These measures do not match exactly onto our notion of 'agency' as conceptualised in this article, but serve adequately as sensitivity measures. Excellent discussions on how these measures differ from our main measure can be found elsewhere (Ibrahim & Alkire, 2007; Lokshin & Ravallion, 2005). First, we assessed impacts on measures of decision-making in large household purchases, food preparation and serving, and women's own pregnancy. We classified respondents into the either 1) *Sole decision-makers* who indicated they are the only one making decisions 2) *Joint decision-makers* who reported making decisions together with others 3) Women who were *not involved, but able to be involved* if they wanted to be involved 4) Women who were *not involved and unable to be involved*, even if they wanted to. In order of empowerment, we ranked women thus: 1 (most empowered), 2, 3 and 4 (least empowered).

Second, we used a measure of perceived ability to affect one's life (Ibrahim & Alkire, 2007), where women were first asked to state what changes to their life, if any, they currently desired following by a question on who would be the most important agent in bringing

about that change (women could name up to three agents). We classified women into 1) women who felt that *no change was required* in their life 2) women who wanted to change an aspect of their life and *included themselves* as an agent of change 3) women who wanted to change, but *excluded themselves* as an agent of change. We ranked women thus: 1 (most empowered), 2, 3 (least empowered).

Third, we assessed impacts on another measure of perceived ability to influence one's life. In the "Power Ladder" (Lokshin & Ravallion, 2005) question, women were shown a picture of a 10-step ladder and told the bottom represented the most disempowered woman imaginable, while the top represented the most empowered woman imaginable. Women were then asked to place themselves on the ladder.

Creation of a wealth index

We measured socio-economic status using a wealth index by performing a categorical factor analysis a list of household assets, housing characteristics, availability of clean water and sanitation facilities, type of cooking fuel and ownership of land (Filmer & Pritchett, 2001; Kolenikov & Angeles, 2009). Instead of grouping factor scores into quintiles, we retained the continuous factor scores to gain maximum discriminatory power. A Q-Q plot revealed a good fit to a normal distribution.

Bibliography

Filmer, D. & Pritchett, L. H. (2001). Estimating wealth effects without expenditure data - or tears: An application to educational enrollments in states of India. *Demography, 38,* 115-132.

Ibrahim, S. & Alkire, S. (2007). Agency and Empowerment: A Proposal for Internationally Comparable Indicators. *Oxford development studies, 35,* 379-403.

Kolenikov, S. & Angeles, G. (2009). Socioeconomic status measurement with discrete proxy variables: Is principal component analysis a reliable answer? *Review of Income and Wealth, 55,* 128-165.

Lokshin, M. & Ravallion, M. (2005). Rich and powerful?: Subjective power and welfare in Russia. *Journal of economic behavior & organization, 56,* 141-172.

Web Table

Table W1. Statements for the main empowerment module. Total scores for external motivation, internal motivation and agency freedom are computed for each domain separately. Agency freedom is calculated as internal motivation – external motivation.

		**Scoring**	
Questions	*Internal motivation*	*External motivation*	*Agency Freedom*
**Domain: Work outside the home**<br>What type of work are you normally involved in?<br>(Work inside the home, work outside the home or both)<br>[If she performs work outside] What type of work outside the home are you normally involved in? (Daily paid labour, regular job, farming, etc.)<br><br>[If the respondent performs work outside] Please indicate whether you agree or disagree with the following statements by saying "yes" or "no":			
a) You do this work because you will get in trouble if you don't.	0	+1	-1
b) You do this work because you want to.	+1	0	+1
c) You do this work because that is what your family members tell you to do.	0	+1	-1

d) You do this work because you personally think it is the right thing to do, whether or not your family members agree.	+1	0	+1
e) You do this work because you like it.	+1	0	+1
f) You do this work so that your family members won't get angry with you.	0	+1	-1
g) You do this work because you want your family members to like you.	0	+1	-1
h) You do this work because it is personally important to you.	+1	0	+1
[If the respondent does not perform work outside] Please indicate whether you agree or disagree with the following statements by saying "yes" or "no":			
a) You do not work outside the home because you will get in trouble if you do.	0	+1	-1
b) You do not work outside the home because you do not want to.	+1	0	+1
c) You do not work outside the home because your family members tell you not to do so.	0	+1	-1
d) You do not work outside the home because you personally think it is the right thing to do, whether or not your family members agree.	+1	0	+1
e) You do not work outside the home because you don't like it.	+1	0	+1
f) You do not work outside the home because your family members might get angry with you if you do.	0	+1	-1
g) You do not work outside the home because you want your family members to like you.	0	+1	-1
h) You do not work outside the home because it is not personally important to you.	+1	0	+1

Questions	**Scoring**<br>*Internal motivation*	*External motivation*	*Agency Freedom*
**Domain: Household chores**			
In your household, who does each of these things most of the time: *Preparing and cooking the main meal, shopping and bringing food for the household, cleaning the home, cleaning dishes and doing laundry, doing household repairs, looking after household money and paying bills, spending time with children and caring for them when they are ill, looking after elderly or ill people, teaching children good behaviour*			
[Referring to the activities that the respondent is engaged in] Please indicate whether you agree or disagree with the following statements by saying "yes" or "no":			
a) You do these activities because you will get in trouble if you don't.	0	+1	-1
b) You do these activities because you want to.	+1	0	+1
c) You do these activities because that is what your family members tell you to do.	0	+1	-1
d) You do these activities because you personally think it is the	+1	0	+1

right thing to do, whether or not your family members agree.			
e) You do these activities because you like doing them.	+1	0	+1
f) You do these activities so that your family members won't get angry with you.	0	+1	-1
g) You do these activities because you want your family members to like you.	0	+1	-1
h) You do these activities because it is personally important to you.	+1	0	+1
**Domain: Health-seeking behaviour**			
Where would you go if you had simple health problems, such as a severe headache or a painful tooth ache? (*A public sector health institution, private medical centre, pharmacy/medical shop, other, would not do anything*)			
[If the respondent answers 'would not do anything'] Please indicate whether you agree or disagree with the following statements by saying "yes" or "no":			
a) You don't do anything because you will get in trouble if you do.	0	+1	-1
b) You don't do anything because you don't want to do anything.	+1	0	+1
c) You don't do anything because that is what your family members tell you to do.	0	+1	-1
d) You don't do anything because you personally think it is the right thing to do whether or not your family members agree.	+1	0	+1
e) You don't do anything because you don't like doing anything.	+1	0	+1
f) You don't do anything so that your family members won't get angry with you.	0	+1	-1
g) You don't do anything because you want your family members to like you.	0	+1	-1
h) You don't do anything because it is not personally important to you.	+1	0	+1

	Scoring		
Questions	*Internal motivation*	*External motivation*	*Agency Freedom*
[If the respondent does not answer 'would not do anything'] Please indicate whether you agree or disagree with the following statements by saying "yes" or "no":			
a) You seek health care the way you do because you will get in trouble if you don't.	0	+1	-1
b) You seek health care the way you do because you want to.	+1	0	+1
c) You seek health care the way you do because that is what your family members tell you to do.	0	+1	-1
d) You seek heath care the way you do because you personally think it is the right thing to do, whether or not your family members agree.	+1	0	+1
e) You seek health care the way you do because you like doing it more than other alternatives.	+1	0	+1

f) You seek health care the way you do so that your family members won’t get angry with you	0	+1	-1
g) You seek health care the way you do because you want your family members to like you.	0	+1	-1
h) You seek health care the way you do because it is personally important to you.	+1	0	+1

Domain: Group participation

Do you participate in any group, organisation, network, association, etc.?

[If the respondent participates in groups] What kind of organisations do you belong to? (*relates to main economic activity, deals with finance, credit or savings, deals with health issues, deals with education issues, political groups or associations, religious groups or associations, ethnic groups or associations, other*)

[If the respondent participates in groups] Which is the most important group or association that you belong to?

[If the respondent participates in groups] Please indicate whether you agree or disagree with the following statements by saying "yes" or "no":

a) You participate because you will get in trouble if you don’t.	0	+1	-1
b) You participate because you want to participate.	+1	0	+1
c) You participate because that is what your family members tell you to do.	0	+1	-1
d) You participate because you personally think it is the right thing to do, whether or not your family members agree.	+1	0	+1
e) You participate because you like it.	+1	0	+1
f) You participate so that your family members won’t get angry with you.	0	+1	-1
g) You participate because you want your family members to like you.	0	+1	-1
h) You participate because it is personally important to you.	+1	0	+1

Questions	Scoring<br>*Internal motivation*	 *External motivation*	 *Agency Freedom*
[If the respondent does not participate in groups] Please indicate whether you agree or disagree with the following statements by saying "yes" or "no":			
a) You don’t participate because you will get in trouble if you do.	0	+1	-1
b) You don’t participate because you don’t want to.	+1	0	+1
c) You don’t participate because your family members tell you not to.	0	+1	-1
d) You don’t participate because you personally think it is the right thing to do, whether or not your family members agree.	+1	0	+1
e) You don’t participate because you don’t like participating.	+1	0	+1
f) You don’t participate so that your family members won’t get angry with you.	0	+1	-1

g) You don't participate because you want your family members to like you.	0	+1	-1
h) You don't participate because it is not personally important to you.	+1	0	+1
